# A new certified reference material for size and shape analysis of nanorods using electron microscopy

**DOI:** 10.1007/s00216-020-02984-z

**Published:** 2020-10-13

**Authors:** Vikram Kestens, Tsvetelina Gerganova, Gert Roebben, Andrea Held

**Affiliations:** 1 European Commission, Joint Research Centre (JRC), 2440 Geel, Belgium; 2 Present Address: European Commission, EUROSTAT, 2920 Luxembourg, Luxembourg; 3 Present Address: European Commission, Directorate-General for Internal Market, Industry, Entrepreneurship and SMEs, 1049 Brussels, Belgium

**Keywords:** Certified reference material, Electron microscopy, Nanorods, Particle shape, Particle size, Titanium dioxide

## Abstract

A new certified reference material (CRM) for size and shape analysis of elongated nanoparticles has been developed by the European Commission’s Joint Research Centre. The CRM consists of titanium dioxide nanorods dispersed in 1-butanol, was coded ERM-FD103 and has been certified for different electron microscopy–based operationally defined measurands such as the modal and median values of the particle number-weighted distributions of the minimum and maximum Feret diameter, the maximum inscribed circle diameter, the area-equivalent circular diameter and the aspect ratio. The nanorods have nominal dimensions of 15 nm in width and 55 nm in length. Homogeneity and stability measurements were performed using transmission electron microscopy. The relative standard uncertainty for homogeneity ranged from 0.3 to 1.7%. No significant instability was detected for a shelf life of 18 months and a storage temperature of 18 °C. The certified values have been determined from the results of an interlaboratory comparison in which qualified expert laboratories participated with scanning and transmission electron microscopy. The certified values are traceable to the unit of length in the International System of Units, the metre, and the relative expanded uncertainties (confidence level of approximately 95%) range from 4 to 6%. These properties allow the CRM to be used for quality assurance and calibration of electron microscopy methods for nanoparticle size and shape analysis in ranges relevant for the implementation of EU legislation related to nanomaterials. The presented study discusses the purpose and results of the different steps that were followed to turn an industrially relevant raw titanium dioxide nanorod material into a fit-for-purpose CRM.

Graphical abstract

**Electronic supplementary material:**

The online version of this article (10.1007/s00216-020-02984-z) contains supplementary material, which is available to authorized users.

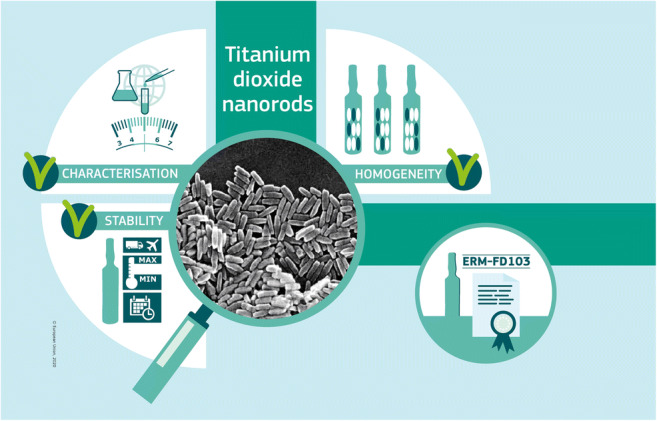

## Introduction

Nanoparticles, as well as materials containing them, are widely used in many applications and products because of their desirable unique chemical and physical properties. Their increased use, and the resulting higher likelihood of exposure, has led to concerns about the potential nano-specific impact [1, 2]. In the European Union (EU), legislation has been put in place to safeguard human health and the environment [3–5]. The nano-specific provisions laid down in the referred horizontal and sectoral legislation are based on the European Commission’s Recommendation (2011/696/EU) on the definition of nanomaterial [6]. The core part of this definition states that “nanomaterial means a natural, incidental or manufactured material containing particles, in an unbound state or as an aggregate or as an agglomerate and where, for 50% or more of the particles in the number size distribution, one or more external dimensions is in the size range 1–100 nm”. Although the definition is overarching in nature, a practical and meaningful implementation requires specifically validated experimental procedures that allow measuring the size of the constituent particles’ minimum external dimensions [7].

Over the last decades, significant advances have been made in the development and optimisation of new and existing techniques for size analysis of (nano)particles [8]. Most of these techniques do not directly measure *true* particle size but, instead, infer size from results obtained for other particle-related physical properties such as sedimentation times or diffusion rates [9]. The particle diameter calculated from such indirect measurements is referred to as an *equivalent* diameter. Unless the particles have a perfectly spherical shape, equivalent diameters obtained from various techniques can differ significantly due to different physical measurement principles and data analysis strategies [10]. Despite these intrinsic differences, the many interlaboratory comparison studies that demonstrated the efficacy and accuracy of popular particle size analysis techniques almost exclusively used (near) spherical particles [11–14]. On the other hand, the concepts of operationally defined measurands and equivalent diameters are not valid for particles of irregular shapes or for agglomerates, as the significance of the diameter deteriorates [15]. When dealing with the implementation of the Commission’s nanomaterial definition, the use of analytical procedures that determine equivalent diameters rather than true minimum external dimensions can lead to biased results and inconclusive assessments.

Compared with those techniques that determine equivalent diameters, and with respect to the implementation of the Commission’s nanomaterial definition, measurement procedures based on scanning and transmission electron microscopy (SEM and TEM) have shown clear superiority as they are capable of counting particles and, simultaneously, measuring their external dimensions [16, 17]. TEM has also proven to be effective for measuring the size of the constituent particles in agglomerates and aggregates [18].

When particle size data are to be used for regulatory purposes and international commerce, then it is of utmost importance that these data are trustworthy and accurate. Confidence in measurement results can be achieved and demonstrated by establishing a reference measurement system around the measurement procedure. For particle size analysis—as in any other measurement field—such system should be based on quality assurance systems such as, or inspired by, ISO/IEC 17025 [19], harmonised and validated measurement procedures (also often called *methods*), and the use of fit-for-purpose certified reference materials (CRMs). According to ISO Guide 30, *reference material* (RM) is the generic term for a group of materials whose homogeneity and stability have been demonstrated and assessed with respect to one or more specified properties [20]. CRMs distinguish themselves from (non-certified) RMs as they have been additionally characterised by a so-called metrologically valid procedure, and are accompanied by a certificate stating the certified value(s) and associated uncertainty(ies) of the specified property(ies), and a statement of metrological traceability [20]. A certified value is a best estimate of the true value. Consequently, CRMs are intended primarily for assessing the accuracy of analytical methods and, in certain cases, for calibration purposes. On the other hand, non-certified RMs are not sufficiently characterised and their metrological applicability is, as a result, limited to quality control applications based on precision assessments.

Both standardised measurement procedures for electron microscopy developed by, for instance, ISO/TC 24/SC 4 [21, 22] and ISO/TC 229 [23, 24], as well as CRMs mainly consisting of (near) spherical nanoparticles [25] are available. These idealised CRMs have been extremely useful during the initial development and validation of particle size analysis methods. However, to comply with sectoral legislation that is based on the Commission’s nanomaterial definition, there is a need for non-spherical nanoparticle CRMs with certified values for well-defined external particle dimensions and shape.

In this contribution, we discuss the development and certification of a titanium dioxide nanorod certified reference material, ERM-FD103, using size and shape measurement results obtained by electron microscopy [26]. A TiO_2_ starting material, synthesised specifically as a candidate RM by an external manufacturer, was processed (i.e. mixing and bottling) and gradually turned into a material with demonstrated and quantified homogeneity and stability. In a next step, the processed candidate CRM was characterised for different particle size and shape measurands through an interlaboratory comparison study. Amongst other, the measurands, or the quantities intended to be measured, included the median value of the distributions of the minimum Feret and maximum inscribed circle diameters, as well as the aspect ratio (i.e. width-to-length ratio). The former two can be directly linked to the Commission’s Recommendation 2011/696/EU on the definition of nanomaterial [6] while aspect ratio is particularly relevant for the implementation of the recently amended REACH Annexes [5], as shape is one of the information requirements for the identification of a nanoform to be registered. In addition to the shape-related measurands, the CRM also embodies a certified value for the area-equivalent circular diameter. This measurand is related neither to the actual external dimensions nor to the shape of the nanorods. However, the equivalent diameter may act as a basic reference for comparing with equivalent diameters from other techniques.

The developed CRM is not the very first of its kind. For instance, a suit of seven gold nanorod reference materials coded as GBW(E)130407-GBW(E)130409 and GBW(E)130474- GBW(E)130477, and certified for their longitudinal surface plasmon resonances and aspect ratios, has been produced by the National Center for Nanoscience and Technology (Beijing, CN). Although these materials can serve particular applications because of their specific optical properties, they lack certified values for external particle dimensions. Moreover, compared with industrially relevant metal oxides such as TiO_2_ and SiO_2_ particles, gold particles exhibit much higher signal-to-noise ratios making them generally easier to analyse. All together, gold nanorod CRMs have thus limited relevance when it comes to quality assurance and validation of routine methods for particle size and shape analysis of industrial materials, which are more challenging to analyse than model particles such as gold. Meanwhile, titanium dioxide has also obtained toxicological interest since it was flagged as potential carcinogenic and mutagenic substance by the French Agency for Food, Environment and Occupational Health & Safety, which successfully submitted TiO_2_ for inclusion in the Community rolling action plan (CoRAP) of the EU [27].

## Materials and methods

### Processing and initial screening

The TiO_2_ nanorod starting material for ERM-FD103 was prepared by nanoComposix (San Diego, USA). The nanorods, which were synthesised from titanium (IV) tert-butoxide using a proprietary sol-gel procedure, were produced as five separate batches of about 0.8 L each and a nominal TiO_2_ mass fraction of 1 g/kg [26]. Each batch was washed multiple times with 1-butanol to reduce the amount of residual surfactant used for the chemical reactions.

After receiving the five batches from the manufacturer, the possible batch-to-batch variation (in terms of nanorod length and width) was preliminarily investigated by means of TEM. Three TEM specimens were prepared from each batch by bringing 15 μL of the as-received suspension onto Alcian blue–treated pioloform- and carbon-coated 400 mesh copper grids (Agar Scientific, Essex, UK) according to a protocol described by Mast et al. [28]. The prepared grids were examined using a Tecnai G2 Spirit microscope (FEI Company, Eindhoven, NL) operated in the bright field mode at 120 kV and a magnification of × 30,000. Micrographs were acquired using a 4 × 4 k Eagle CCD camera (FEI Company). For each prepared specimen grid, 250 non-overlapping nanorods were analysed for their minimum Feret (*F*_min_) and maximum Feret (*F*_max_) diameters using the iTEM software (Olympus, Münster, DE). The TEM experiments were conducted by an external laboratory.

After acceptance, the five batches were further processed at the Joint Research Centre (JRC, Geel, BE). This included first combining the volumes of the five as-received TiO_2_ batches in a clean glass bottle, followed by flushing with argon and overnight mixing with a magnetic stirrer. Before filling, the 5 mL glass ampoules (Nederlandse Ampullenfabriek B.V., Nijmegen, NL), which were chosen as a rugged and gas tight sample containment, were opened, rinsed with purified water and dried in an oven. The cleaned and dry ampoules were then manually loaded on the feeding belt of a Rota ampouling machine R 90 PA (Rota, Wehr, DE) where they were subsequently flushed with Ar gas, filled with approximately 2 mL of suspension, again purged with Ar gas, flame sealed, and finally labelled with an indication of the CRM code (i.e. ERM-FD103) and an individual identification number. A total of 1500 ampoules were produced and temporarily stored at 4 °C until the optimal storage temperature (i.e. 4 °C or 18 °C) was determined.

### Homogeneity testing

The aim of a homogeneity study is to assess the *between-unit* homogeneity, i.e. this is the estimated variation of the certified properties/measurands between the different units of a CRM batch, and to demonstrate that all produced units are either identical or that differences are negligibly small [29]. To be able of detecting potentially small but significant differences, ISO 17034 requires that the measurements of a homogeneity study are performed with a method with constant bias and appropriate precision (i.e. high repeatability). For this reason, measurements are preferably conducted by a single laboratory and under repeatable conditions.

The homogeneity between the units of ERM-FD103 was assessed according to ISO Guide 35 [30]. Nineteen units were selected from the processed batch using a so-called random stratified sampling scheme. This sampling scheme consisted of dividing the batch into 19 sub-groups (with a similar number of units in each sub-group) and then randomly selecting one unit from each sub-group. Finally, 15 out of the 19 selected units were analysed during the homogeneity study. The remaining four units were considered as backup only.

The homogeneity study was based on TEM measurements performed by an external expert laboratory. Prior to specimen preparation, each unit was diluted five times in 1-butanol as to obtain a suitable concentration and spread of the particles onto the grid surface. The optimal dilution factor was determined after investigating a series of concentrations. Then, 15 μL of the diluted suspension was brought onto pioloform- and carbon-coated copper grids (Agar Scientific, Essex, UK) and left in contact for 10 min. After 10 min, the excess suspension was carefully removed with a filter paper and the grids were allowed to air dry at room temperature. Two independent grids were prepared from each unit. During specimen preparation, the grids were kept in closed Petri dishes to reduce fast evaporation of the dispersant and as a protection against airborne dust particles. Experiments were performed using a Tecnai G2 Spirit transmission electron microscope (FEI Company, Eindhoven, NL) operated in the conventional bright field mode at an acceleration voltage of 120 kV and a magnification of × 30,000. Micrographs were acquired using a 4 × 4 k Eagle CCD camera (FEI Company). For each specimen, at least 500 non-overlapping particles were quantitatively analysed for *F*_min_, *F*_max_ and area-equivalent circular diameter (ECD) using the AnalySIS Solution of the iTEM software (Olympus, Münster, DE). To be able to separate potential systematic drift in the analytical sequence from potential trends in the ampoule filling sequence, measurements were performed in a randomised manner.

The between-unit homogeneity of ERM-FD103 was assessed by evaluating the modal and median results acquired for *F*_min_, *F*_max_ and ECD using regression analysis and Grubbs’ outlier testing at 95% and 99% confidence levels, respectively. ISO 17034 requires assessment of the homogeneity (and stability) of the measurands of all certified values. As it was considered that monitoring *F*_min_ and *F*_max_ should be sufficient to capture any potential homogeneity (and stability) issue that may occur to the nanorods, the maximum inscribed circle diameter was not evaluated during the homogeneity (and stability) study.

Because the preparation of TEM test specimens and image acquisition are time-consuming, experiments were spread over eight different days. Using single-factor analysis of variance (ANOVA), the between-unit variation (*u*_bb_) was quantified as described by Linsinger et al. [31]. For nanorods, *F*_min_ and the maximum inscribed circle diameter probe the same external dimension. Therefore, the uncertainty estimated for *F*_min_ was used also for the maximum inscribed circle diameter.

In case of a significant trend in the ampoule filling sequence, the standard uncertainty associated to a rectangular distribution (*u*_rec_) between the highest and lowest unit mean (Eq. 1) was taken as alternative for *u*_bb_:

1 $$ {u}_{\mathrm{rec}}=\frac{\left|\mathrm{highest}\ \mathrm{mean}-\mathrm{lowest}\ \mathrm{mean}\right|}{\sqrt{3}\ \overline{y}} $$

where $\overline{y}$ is the mean of all results for the given measurand.

### Micro-homogeneity testing

In addition to the between-unit homogeneity, also the homogeneity within each unit is a critical requirement for any reference material. The latter, also referred to as micro-homogeneity, defines the minimum sample amount to be taken from a CRM unit without compromising the sample’s representativeness. The minimum volume to be sampled from an as-received and undiluted unit of ERM-FD103 was determined from the results and method information of the characterisation study. The smallest sample intake used by a laboratory in the characterisation study, and that still yielded results with acceptable accuracy, was considered as minimum sample intake for ERM-FD103.

For electron microscopy–based measurement procedures, which are known to be time-consuming and expensive, the minimum number of particles to be analysed per specimen is an important parameter to ensure statistical accuracy with a minimum of measurement effort. The assessment of the minimum number of particles was based on simulating particle size distributions for *F*_min_, *F*_max_ and ECD using 100, 200, 250, 300, 500 and 1000 particles, respectively, that were selected randomly from a total of 33,526 particles that were analysed previously for between-unit homogeneity. For each distribution, the 33,526 particles were randomised in Excel using the (=RAND) function, after which the 100, 200, and so forth, first listed particles were selected. The corresponding particle size results were then classified in bins of 1 nm in width and plotted as histograms covering the size range of 1 to 100 nm. The histograms were fitted with a Gaussian function using the Levenberg Marquardt iteration algorithm (OriginLab Corporation, MA, USA). From the Gaussian fit, the modal value and the full-width at half maximum (FWHM) were determined and its ratio (FWHM/mode) was used as a normalised indicator for the distributions’ degree of polydispersity. For each measurand, the randomisation, particle selection and data fitting process were performed in six-fold to allow defining the minimum required number of particles statistically using single-factor ANOVA and the Cochran’s outlier test, according to ISO 5725-2 [32], at a confidence level of 95%.

### Stability testing

Reference materials must be stable, with respect to their certified properties, when being transported to customers (cf. short-term stability) and during storage (cf. long-term stability) [29]. The short- and long-term stability of ERM-FD103 was assessed according to requirements described in ISO Guide 35 [30]

**Fig. 1:**
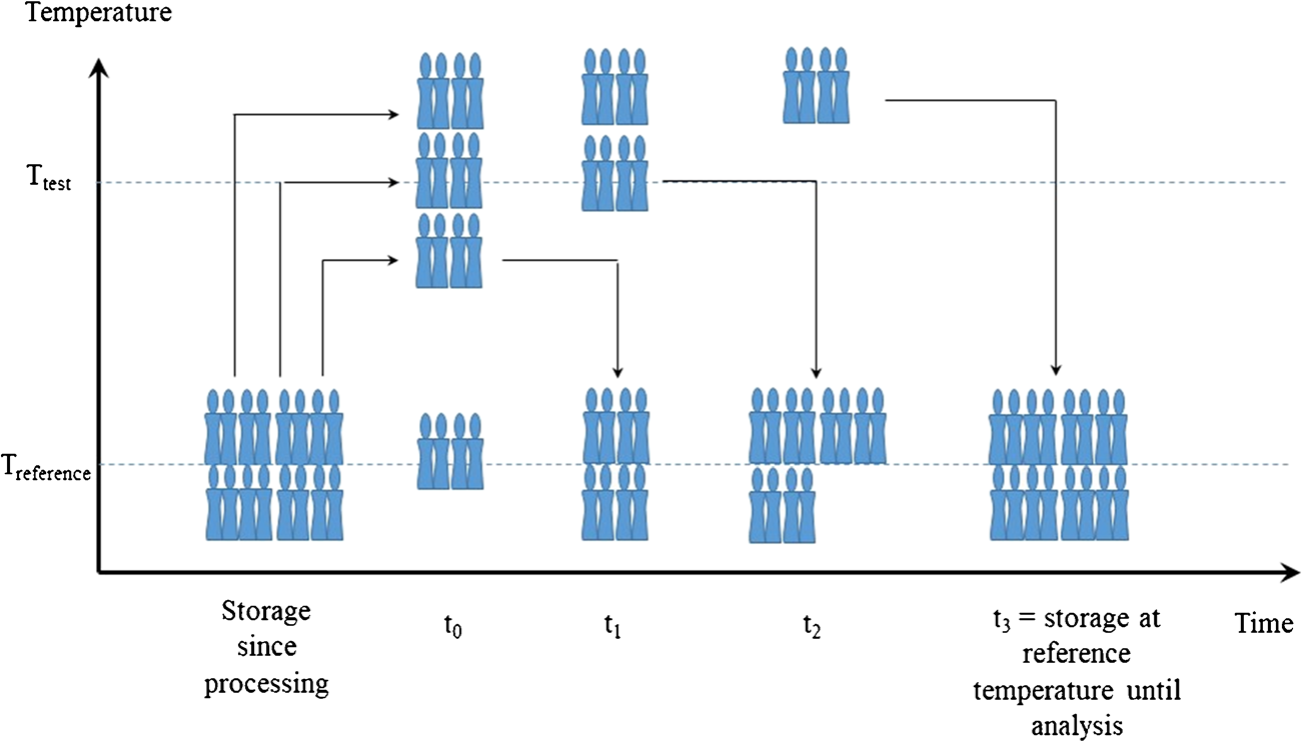
Schematic illustration of an isochronous stability study indicating the systematic shift of a selection of sample units according to predefined temperatures and exposure times (after Figure 4 in the article [34]). *T*_reference_ temperature at which the CRM is expected to be stable, *T*_test_ temperature for which stability is investigated, *t*_0_, *t*_1_, *t*_2_ and *t*_3_ time points corresponding to different time intervals during which units are kept at a specific temperature

The short-term stability of ERM-FD103 was examined at two temperatures (4 °C and 60 °C) and three time intervals (1, 2 and 4 weeks) following an isochronous design [33]. The test temperatures and exposure times were chosen such as to mimic realistic transport conditions. The reference temperature was set to 18 °C (time point 0) assuming that no significant changes in physico-chemical properties occur at room temperature and during the relatively short time lapse of the study. It must be noted that a reference temperature of 18 °C is rather unusual as for most types of environmental and biological matrix CRMs, for instance, certified for the elemental composition of contaminants, a reference temperature of − 20 °C is commonly used as to virtually *freeze* any potential decomposition process. For colloids, it is however known that a low thermal energy can induce particle agglomeration processes. The latter is unwanted for colloidal CRMs intended for particle size analysis. The isochronous study involved moving all units, that were selected using a stratified random sampling scheme, simultaneously to the respective test temperature and systematically moving the units back to the reference temperature at 18 °C after respectively 1, 2 and 4 weeks. The units were then kept at 18 °C prior to analysis. For each temperature and time point, four units were analysed in duplicate and in a randomised sequence for *F*_min_ and *F*_max_ using the TEM procedure described previously (see homogeneity testing). The concept of an isochronous study is schematically presented in Fig. 1.

During transportation, samples may be exposed to sub-zero centigrade temperatures. Although the TiO_2_ nanorods are dispersed in 1-butanol, which has a freezing point of about − 89 °C, the possible effect of 1 week at − 20 °C was additionally investigated by storing two units for 1 week at − 20 °C. Results were compared with results obtained for units stored at the reference temperature.

Immediately after processing, the 1500 sample units were temporarily stored at 4 °C. However, long-term storage at room temperature is more practical and reduces storage costs. In that respect, a storage temperature of 18 °C was envisaged in the long-term stability study. In addition, a study at 60 °C was run in parallel to investigate the stability (or instability) of the material when exposed to extreme conditions. In both cases, units were again selected from the batch using stratified sampling and stored at the respective test temperature for 4, 8 and 12 months. In contrast to the short-term stability study, the reference temperature was this time set to 4 °C instead of 18 °C, as the test temperature and reference temperature cannot be the same. For both temperature regimes, two units per time point were analysed in duplicate using TEM and again applying an isochronous scheme that followed the generic concept as illustrated in Fig. 1.

For most measurands, the relative standard uncertainties, *u*_lts_, estimated for a shelf life of 12 months exceeded the predefined maximum uncertainty of 2%. Therefore, a second isochronous study was run with a maximum time point at 27 months using four units that were stored at 18 °C and two units from the reference batch that was kept at a temperature of 4 °C. These units were analysed again in duplicate by an external laboratory using TEM. To effectively merge the two isochronous datasets of the different measurands, the 12 replicate results of the second isochronous study were normalised against the mean value calculated from the 28 replicate results of the first long-term stability study, thereby bringing both datasets to a notionally common scale. After normalisation, the datasets were pooled together, yielding a total of 40 replicate results. Finally, the 18 °C (up to 27 months) and the 60 °C (up to 12 months) isochronous datasets were evaluated for statistical outliers and trends.

The results obtained for the modal and median values of *F*_min_ and *F*_max_ were screened for statistical outliers using the single and double Grubbs’ test on a confidence level of 99%. For each temperature, curves of storage times vs. measurement results were plotted and the slopes of the regression lines were tested for significance using a two-tailed *t* test at a 95% confidence level. The uncertainty contributions from short-term stability (*u*_sts_) and long-term stability (*u*_lts_) were estimated according to the equations described by Linsinger et al. [31, 35]. The relative standard uncertainties for long-term stability, which were estimated from the product of the chosen shelf life (i.e. 18 months) and the uncertainty of the regression line, included an additional relative uncertainty contribution, *u*_d_, that compensates for the data normalisation (Eq. 2),

2 $$ {u}_{\mathrm{d}}=\sqrt{\frac{1}{n_1}{RSD}_1^2+\frac{1}{n_2}{RSD}_2^2} $$

where *RSD*_1_ and *RSD*_2_ are the relative standard deviations of all results in the first and second isochronous study, respectively, and *n*_1_ and *n*_2_ are the number of data points in the two studies.

### Batch characterisation and assignment of certified values

Following the provisions given in ISO 17034, the assignment of certified values for the selected operationally defined measurands (i.e. the modal and median values of *F*_min_, *F*_max_, maximum inscribed circle diameter, ECD and aspect ratio distributions) was based on a characterisation study that was organised as an interlaboratory comparison (ILC) study amongst expert laboratories [29]. A description of the measurands and simplified representations are given in Table 1. Before inviting laboratories to participate in the ILC, JRC performed a preliminary qualification of candidate laboratories based on documentary evidence of the laboratory’s expertise in the specific measurement field and with quality assurance, e.g. based on ISO/IEC 17025 accreditation.

**Table 1:**
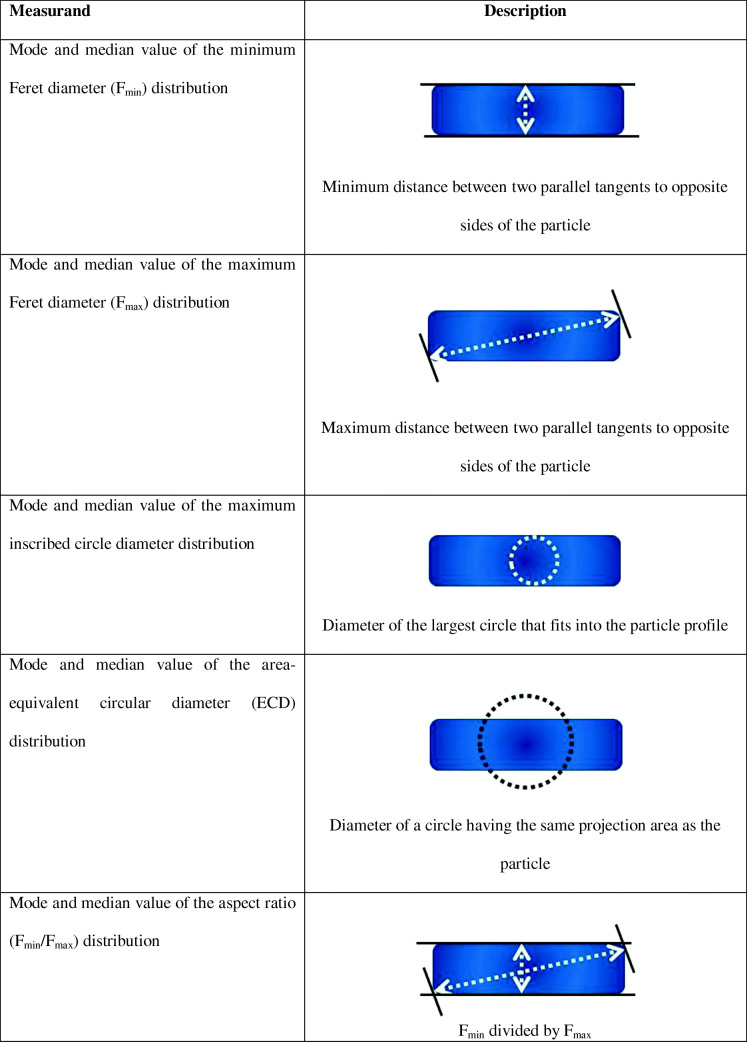
Operationally defined measurands considered for characterisation [21]

Eight laboratories participated in the ILC (Table 2). Two of them participated with both SEM and TEM, one with SEM only and five with TEM only. Each laboratory received three units of ERM-FD103 and one unit of a quality control material (QCM). This QCM was a blinded CRM (i.e. monomodal near-spherical colloidal silica ERM-FD100 [36]) and its results were used by JRC to validate the laboratories’ results obtained on ERM-FD103, i.e. if the results of the QCM significantly differed from the certified value (i.e. 19.4 nm ± 1.3 nm), then, the ERM-FD103 dataset was excluded from the value assignment procedure. The laboratories were instructed to spread the experiments on three different days (one unit/day). The QCM had to be analysed on the first measurement day. Each sample unit had to be measured in duplicate. They were also asked to perform measurements according to their in-house validated measurement procedure while adhering to a common protocol that outlined general instructions regarding specimen preparation, image analysis and reporting (see Electronic Supplementary Material, ESM). A detailed description of the measurement procedures is available in the ESM.

**Table 2:** Qualified expert laboratories participating in ILC study (in alphabetical order)

Laboratory	Country
Agfa Gevaert, Agfa-Labs	Belgium
Evonik Technology & Infrastructure GmbH	Germany
Industrial Technology Research Institute (ITRI)	Taiwan
National Institute of Standards and Technology (NIST)	USA
National Measurement Institute	Australia
MVA Scientific Consultants	USA
Sciensano	Belgium
University of Namur	Belgium

All datasets received from the ILC participants were first checked for completeness and compliance to the ILC protocol. In the next step, the measurement results of the QCM were compared with its certified value and uncertainty according to a procedure described in ERM Application Note 1 [37]. Datasets that were found technically invalid were excluded from the study. The technically valid datasets were tested for normality of dataset means using kurtosis/skewness tests and for outlying means and standard deviations using the Grubbs’ and Cochran’s tests (both on a 99% confidence level). Certified values were calculated as the unweighted mean of the laboratory means of the retained data. The uncertainties of the assigned certified values were estimated by combining the relative standard uncertainties from between-unit homogeneity (*u*_bb_), short-term stability (*u*_sts_), long-term stability (*u*_lts_) and characterisation (*u*_char_). The latter was estimated as the standard error of the mean of laboratory means (Eq. 3):

3 $$ {u}_{\mathrm{char}}=\frac{s}{\sqrt{n}} $$

where *s* is the standard deviation calculated from the laboratory mean results, and *n* is the number of laboratory mean results.

The different relative standard uncertainties were combined using the root-sum-square method (Eq. 4), following guidelines described in ISO/IEC Guide 98-3 [38]. The expanded uncertainty, *U*_CRM_, was calculated by multiplying the combined standard relative uncertainty, *u*_c_, with a coverage factor *k* = 2 (Eq. 5). The coverage factor of 2 was chosen as it defines a confidence level of approximately 95%.

4 $$ {u}_{\mathrm{c}}=\sqrt{u_{\mathrm{bb}}^2+{u}_{\mathrm{sts}}^2+{u}_{\mathrm{lts}}^2+{u}_{\mathrm{c}\mathrm{har}}^2} $$

5 $$ {U}_{\mathrm{CRM}}={u}_{\mathrm{c}}\times k $$

## Results and discussion

### Source material and initial screening

**Fig. 2:**
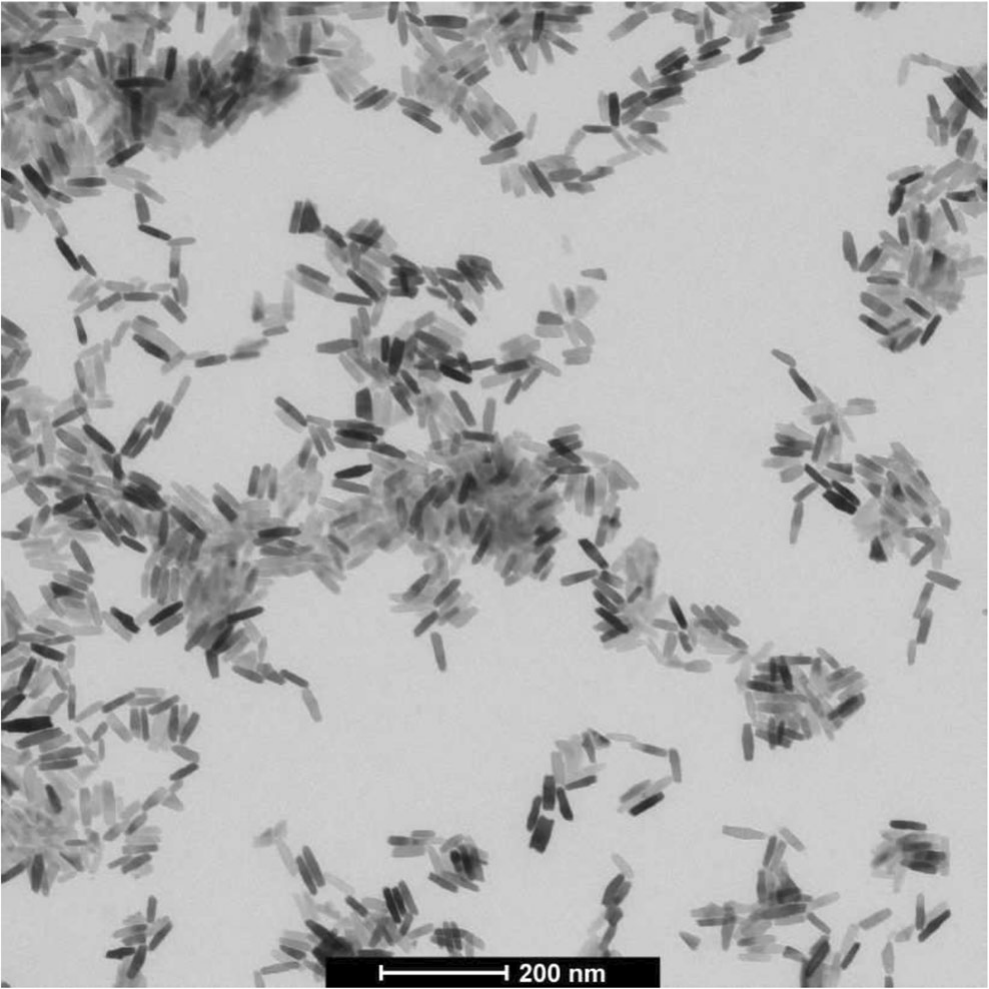
Representative TEM micrograph of ERM-FD103

The main rationale for choosing titanium dioxide particles with elongated shapes as analyte for ERM-FD103 was on the one hand the need for a CRM of non-spherical particles that can support, for instance through quality assurance and method validation, the implementation of the European Commission’s nanomaterial definition [6]. On the other hand, rather than opting for model particles, such as gold nanorods, it was decided to select TiO_2_ as this material poses a bigger analytical measurement challenge and has a greater industrial relevance. Due to its various properties, nanosized titanium dioxide has nowadays become a popular ingredient in many consumer products [39]. Due to the absence of off-the-shelf available raw suspensions of TiO_2_ nanorods with average length and width of (50 ± 10) nm and (15 ± 3) nm, respectively, a suitable source material with tailored properties was synthesised by an external company. The aforementioned predefined external particle dimensions, and their dimensional relationship are not necessary for the material to be a CRM. However, they were chosen as to be sufficiently below the 100 nm size threshold used in the Commission’s nanomaterial definition, and to ensure that both extreme dimensions can be imaged with sufficient resolution at a common magnification. An overview of nominal information on the titanium dioxide nanorod starting material, as provided by the manufacturer, or obtained from preliminary batch characterisation by TEM, is given in Table 3. A representative TEM micrograph is shown in Fig. 2.

**Table 3:** Nominal (non-certified) information on titanium dioxide starting material

Property	Specifications/observations
Appearance of suspension	Milky white
Nominal nanorod length	55 nm^a^
Nominal nanorod width	17 nm^a^
Nominal TiO_2_ mass concentration	1.0 g/L
Particle concentration	3.8 × 10^15^ particles/mL
Dispersant	1-butanol

^a^Preliminary batch characterisation by TEM

### Homogeneity

The between-unit homogeneity of ERM-FD103 was evaluated using results obtained on 15 units that were analysed in duplicate by TEM. Quantitative image analysis was performed for *F*_min_, *F*_max_ and ECD. A description of the measurands and simplified representations are given in Table 1.

**Fig. 3:**
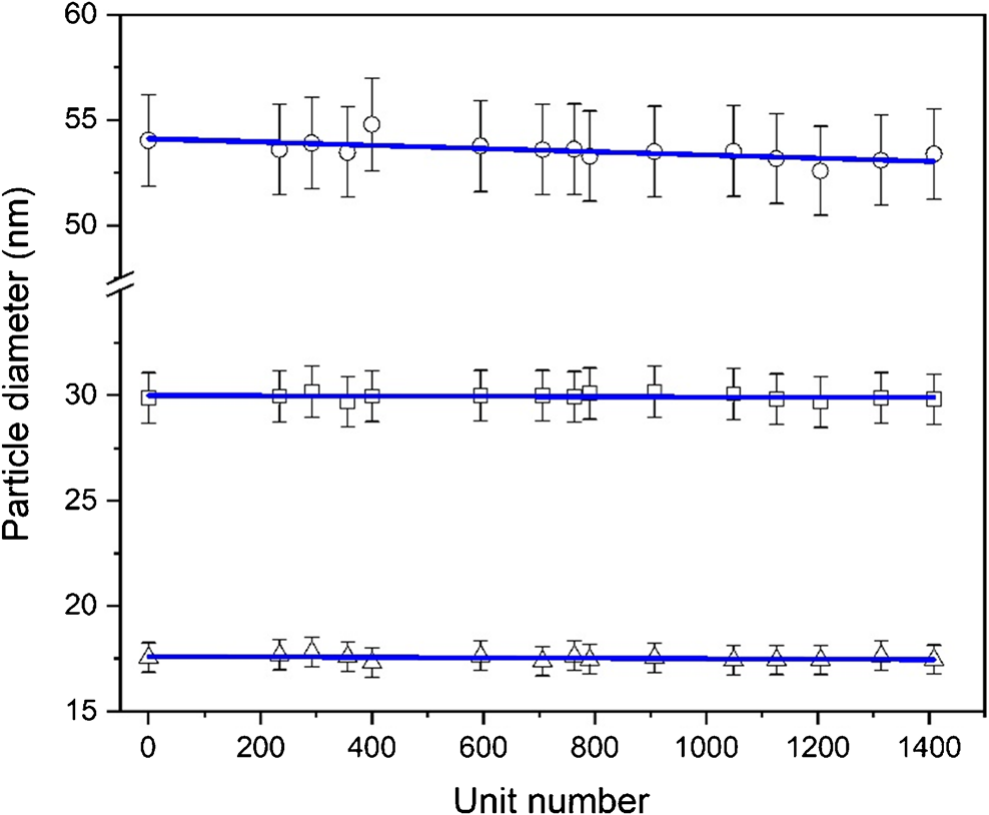
Homogeneity data (average results of modal values of two replicates) for *F*_min_ (triangles), *F*_max_ (circles) and ECD (squares) measurands as determined by TEM with overlaid linear fits; error bars correspond to the expanded measurement uncertainties (*k* = 2)

The results obtained (Fig. 3) were tested using regression analysis and Grubbs' outlier testing. Neither outliers nor significant trends in the ampoule filling or analytical sequence were observed at a confidence level of 95% for *F*_min_ and ECD. As a result, it was possible to further evaluate these results with single-factor ANOVA and estimate the relative standard uncertainties for between-unit homogeneity, *u*_bb_, as in [31]. For both the modal and median values of *F*_max_, the negative slopes of the regression lines were found to be significantly different from zero at a 95% confidence level, indicating a statistically significant trend for *F*_max_ in the filling sequence. While a technical explanation could not be found, the impact of the significant trend on the actual homogeneity of *F*_max_ is very low and considered irrelevant when comparing to the underlying precision uncertainty of 4% (i.e. error bars). To compensate for the statistical trend, a rectangular distribution [38] was used for estimating an alternative relative standard uncertainty, *u*_rec_, for the between-unit homogeneity of *F*_max_. Based on the results of the homogeneity study, it can be concluded that the between-unit variation of the certified measurands is consistent and sufficiently small to ensure equivalence of the 1500 processed units of ERM-FD103.

The relative standard uncertainty for between-unit homogeneity ranged between a minimum of 0.3% for ECD and a maximum of 1.4% for *F*_max_. An overview of the homogeneity uncertainties is given in Tables 4 and 5. Similar results were obtained for the median values, even if median values are statistically less robust than modal values. It must be noted that the absolute values may differ from the certified values due to potential laboratory bias, but this is irrelevant for the evaluation of homogeneity (and stability).

**Table 4:** Uncertainty budget for *F*_min_, *F*_max_, maximum inscribed circle diameter and ECD

Measurand	Certified value (nm)	*u*_bb_ (%)	*u*_sts_ (%)	*u*_lts_ (%)	*u*_char_ (%)	*U*_CRM_ (nm)
*F*_min_ (mode)	16.0	0.6	0.2	1.0	2.5	0.9
*F*_min_ (median)	16.1	0.7	0.2	0.9	2.4	0.9
*F*_max_ (mode)	53.5	1.7	0.1	1.3	1.1	2.6
*F*_max_ (median)	54.0	1.3	0.1	1.3	1.0	2.4
Maximum inscribed circle diameter (mode)	15.1	0.6	0.2	1.0	2.0	0.7
Maximum inscribed circle diameter (median)	15.1	0.7	0.2	0.9	1.8	0.7
ECD (mode)	29.8	0.3	0.2	1.3	1.3	1.2
ECD (median)	29.9	0.3	0.2	1.3	1.6	1.3

**Table 5:** Uncertainty budget for aspect ratio

Measurand	Certified value	*u*_bb_ (%)	*u*_sts_ (%)	*u*_lts_ (%)	*u*_char_ (%)	*U*_CRM_
Aspect ratio (mode)	0.298	0.9	0.3	1.7	2.3	0.018
Aspect ratio (median)	0.296	0.9	0.3	1.6	1.0	0.013

### Micro-homogeneity

The certified values, and their associated uncertainties, of a CRM are only valid if the size of the sample taken from an as-received CRM unit is at least equal to or greater than the *minimum sample intake*. Taking too small sample portions can significantly degrade the repeatability of the measurement. For ERM-FD103, a minimum sample intake of 5 μL was determined. This value was derived from the technically valid laboratory datasets that were used for value assignment and from the information on specimen preparation (see section on batch characterisation and assignment of certified values), as provided by the ILC participants, i.e. 5 μL was the smallest amount of undiluted material sampled by a laboratory (L5-TEM) providing valid data.

When particle-based CRMs are used for measurement procedures based on image analysis, then also the number of particles analysed is a critical parameter as it will determine how well the experimental particle number-based distribution can be expected to describe the actual or true distribution. Expectedly, the more particles counted and analysed the better this agreement will be. However, electron microscopy is an expensive and time-consuming method and the analysis of thousands of particles may not always be affordable. Therefore, determining the minimum number of particles is of key importance.

**Fig. 4:**
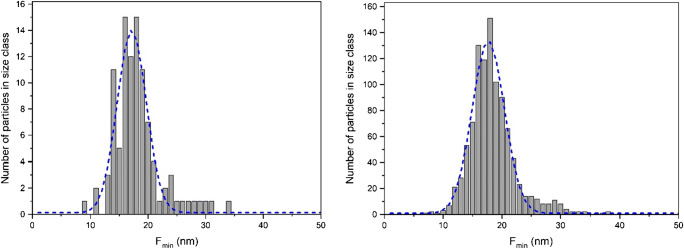
Frequency histograms (grey bars) for *F*_min_ based on 100 (left) and 1000 (right) analysed particles with overlaid Gaussian fits (dashed lines)

**Fig. 5:**
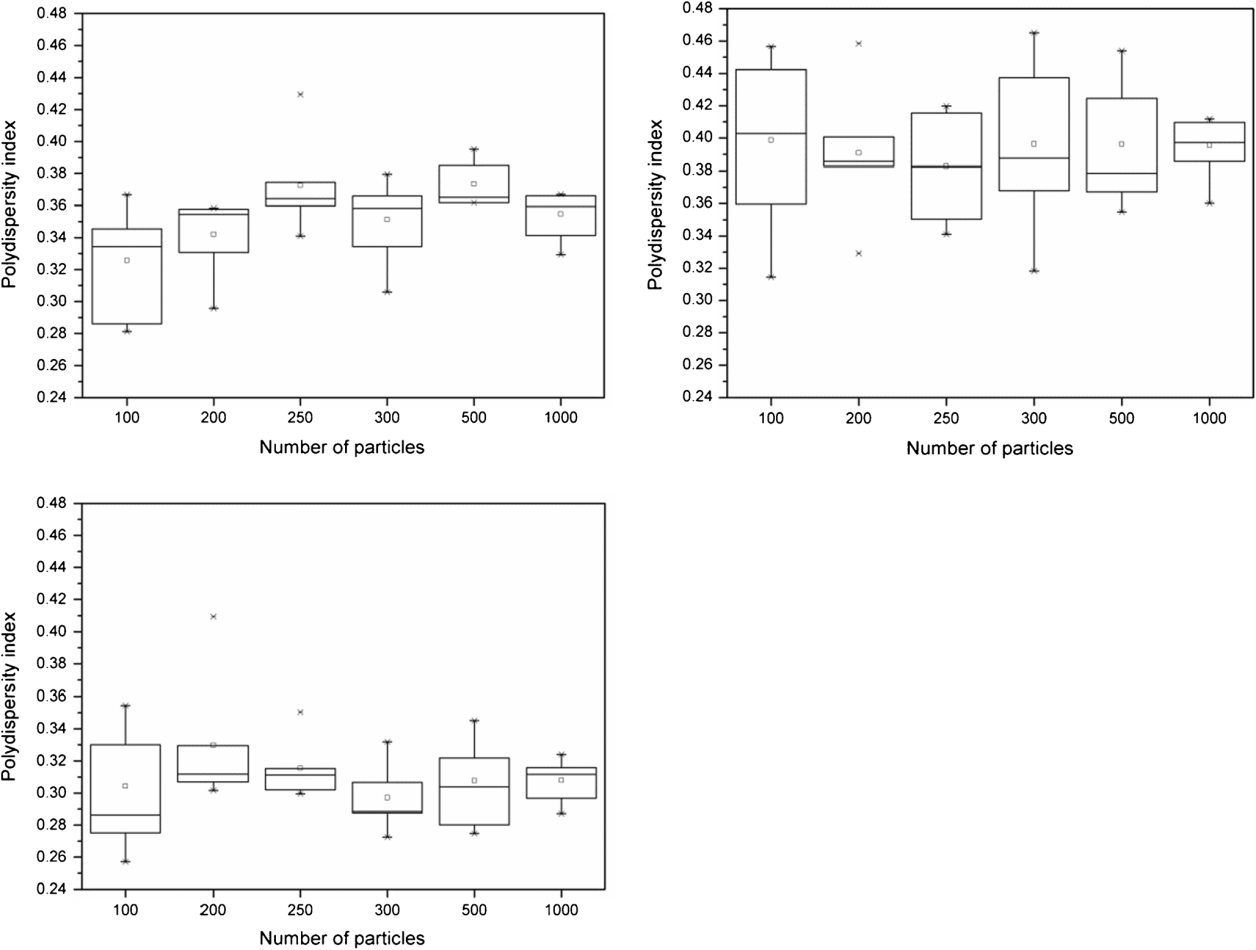
Box and whisker plots of polydispersity indices of *F*_min_ (top left), *F*_max_ (top right) and ECD (bottom left) histograms at different numbers of particles

For ERM-FD103, histograms were computed for different numbers of particles. As an example, typical histograms for *F*_min_ based on 100 particles and 1000 particles, with overlaid Gaussian fits, are shown in Fig. 4. The Gaussian fits were used to determine the FWHM and the modal values of the histograms in a robust manner. It must be noted that lognormal functions are generally recommended for fitting of particle size distributions because they are more flexible and can support heavy tailed distributions. However, Gaussian functions can be used reliably if the distributions are monodisperse and narrow. This was the case for the *F*_min_, *F*_max_ and ECD measurands of ERM-FD103. An overview of the average values calculated from the six replicate results for the relevant peak characteristics (mode, FWHM) determined from the Gaussian fits of the *F*_min_, *F*_max_ and ECD histograms is given in Table 6, the calculated polydispersity indices (PI) are presented as box and whisker plots in Fig. 5. The relative expanded (*k* = 2) measurement uncertainties for the particle size results for *F*_min_, *F*_max_ and ECD are 6%, 5% and 4%, respectively, as estimated by the external laboratory that performed the TEM measurements. Measurement uncertainties are not available for the results of FWHM.

**Table 6:** Average values of peak characteristics (mode, FWHM) determined from Gaussian functions fitted to histograms of *F*_min_, *F*_max_ and ECD measurands

Number of particles	*F*_min_		*F*_max_		ECD	
	Mode (nm)	FWHM (nm)	Mode (nm)	FWHM (nm)	Mode (nm)	FWHM (nm)
100	17.5	5.7	54.5	21.7	30.0	9.1
200	17.8	6.1	54.4	21.3	30.0	9.9
250	17.7	6.6	54.6	20.9	29.7	9.1
300	17.8	6.3	55.1	21.9	30.1	8.9
500	17.7	6.6	54.7	21.7	29.9	9.2
1000	17.6	6.3	54.7	21.6	30.0	9.2

For the three considered operationally defined particle size measurands, single-factor ANOVA was first conducted to test significance of the different *particle number* group means at a confidence level of 95%. For the modal, FWHM and PI results of *F*_max_ and ECD, the null hypothesis (i.e. no significant differences amongst group means) was accepted as both the *F* statistics and the *p* values were always large and above their critical values. Similar statistical results were obtained for the modal value of *F*_min_. However, for the FWHM and PI results of *F*_min_, the *p* value was less than the applicable alpha value of 0.05, thus rejecting the null hypothesis that there are no significant differences between the group means. It can be seen from the results in Table 6 that the significance, flagged by ANOVA, is due to the lower average FWHM value when analysing as few as 100 particles. This observation, however, does not follow a logical pattern, as the width or the dispersal of the data points around the mean is expected to decrease with an increased number of particles. As the *F* statistic from ANOVA can be sensitive to unequal variances, the Cochran’s outlier test was used additionally to closer examine the homogeneity of the variances. Despite the significantly lower group mean, it was found that the corresponding Cochran’s test statistics, as calculated according to the outlier procedures described in ISO 5725-2 [32], were less than the 5% critical values, thus demonstrating homogeneity of the different variances.

Based on the above statistical interpretations, it was concluded that the analysis of 100 particles (individual or touching, but not overlapping) is sufficient for reliably comparing measurement results with the certified particle size and shape values of ERM-FD103.

### Stability

**Fig. 6:**
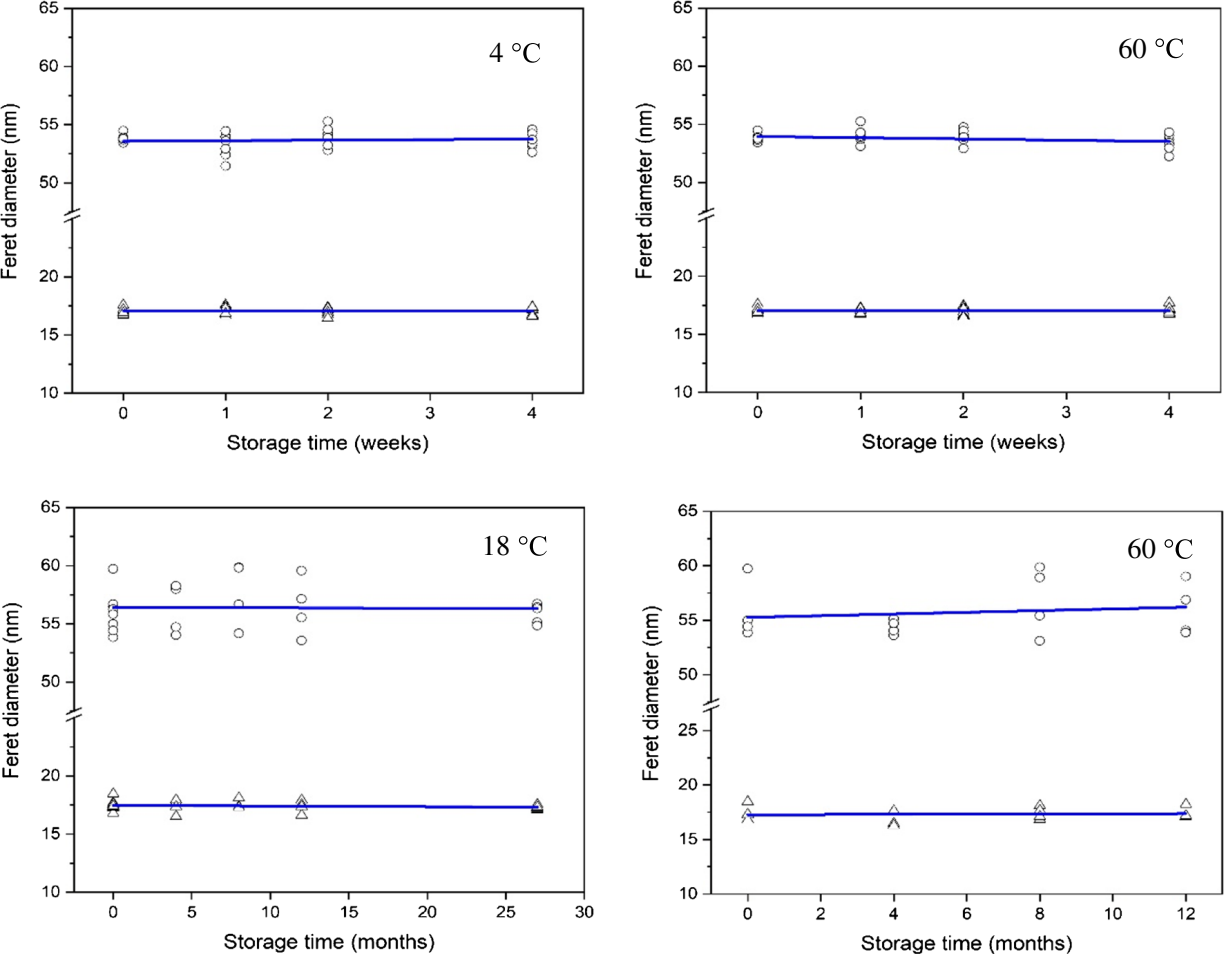
Short-term (top) and long-term (bottom) stability plots of data (average results of modal values of two replicates) for *F*_min_ (triangles) and *F*_max_ (circles) with overlaid regression lines. Individual error bars corresponding to relative expanded uncertainties of 4% are omitted for reasons of clarity

The short-term stability results obtained for the measurands *F*_min_ and *F*_max_ were grouped according to temperature and time point. Using the single and double Grubbs’ tests at a confidence level of 99%, no outlying individual results were found in the data for any storage temperature. The individual data were also plotted (Fig. 6) against their corresponding storage times, and regression lines were fitted to check for significant trends (at a 95% confidence level) indicating possible changes of the measurands with time. None of the slopes was found significantly different from zero. In addition to the storage temperatures of 4 °C and 60 °C, no difference was observed between units stored at − 20 °C and units stored at the reference temperature (data not shown), confirming that no special packaging requirements are needed when shipping units of ERM-FD103 to customers during cold winter months. Supported by the experimental data and taken into account a maximum dispatch of 1 week, it can be concluded that ERM-FD103 can be safely shipped under ambient conditions.

Data from two long-term stability studies were combined to assess the stability of ERM-FD103 when stored at 18 °C. After normalisation, the data of the two studies were pooled together and analysed for outliers and significant slopes. Neither significant outliers nor significant slopes were detected demonstrating that ERM-FD103 can be safely stored at 18 °C. The linear regression line of the results of the 60 °C study shows a slight but systematic increase for the measured values of *F*_max_ with increasing storage time. Based on the overlaid regression line, an increase of about 0.3% can be predicted after 12 months of storage. However, since the slope was found insignificant when compared with the relative expanded uncertainty of 4% of the size values, it can be concluded that ERM-FD103 is also sufficiently stable even at higher temperatures and for at least 12 months. In addition, after the certification study, ERM-FD103 will be included in JRC’s regular stability monitoring programme, to monitor its further stability.

### Batch characterisation and assignment of certified values

The characterisation of ERM-FD103 was based on an interlaboratory comparison (ILC) study in which eight expert laboratories participated with SEM- and/or TEM-based methods. The laboratories were encouraged to use their own in-house validated SEM- and/or TEM-based measurement procedure while following some general but detailed instructions regarding sample handling, sampling, specimen preparation, image analysis and reporting (see ESM). The decision to allow the laboratories to use their own analytical measurement procedures, instead of providing all laboratories with a common and detailed procedure, increases the versatility and the robustness of the certified values, eventually assigned to ERM-FD103. A summary of the key method parameters and their corresponding conditions, used by the ILC participants, is given in Table 7. A more complete description of the laboratories’ measurement procedures is available in Table S1 (see ESM). These measurement procedures may be a source of information for other laboratories in need of developing an SEM or TEM method for analysing the size and shape of nanoparticles.

**Table 7:** Key parameters and conditions of measurement procedures used by participants of the ILC study

Method parameter	L1-TEM	L2-TEM	L3-TEM	L4-SEM	L5-TEM	L6a-TEM	L6b-SEM	L7a-TEM	L7b-SEM	L8-TEM
Instrument	FEI Tecnai Spirit	Philips CM120	Hitachi H-7500	FEI Helios Dual-Beam	Philips CM200	FEI Tecnai G2	JEOL 7500F	JEOL JEM-2100F	JEOL JSM-6500F	JEOL 2100
Sample volume (μL)^a^	15	10	200	NA^b^	5	100	100	800	800	NA^b^
Acceleration voltage (kV)	120	100	100	15	80	200	15	200	15	200
Magnification	× 30,000	NA^b^	× 8000	× 300,000	× 150,000	× 390,000	× 200,000	× 40,000	× 120,000	× 56,000
Pixel size (nm)	0.37	0.42	3.12	0.6	0.37	0.14	0.47	0.41	0.78	0.26
Min. number of particles^c^	588	502	280	550	256	501	261	284	512	473

^a^Volume taken from the as-received sample unit, either brought onto a specimen substrate or further diluted

^b^Information not provided by ILC participant

^c^Minimum number of particles analysed for a specimen

**Fig. 7:**
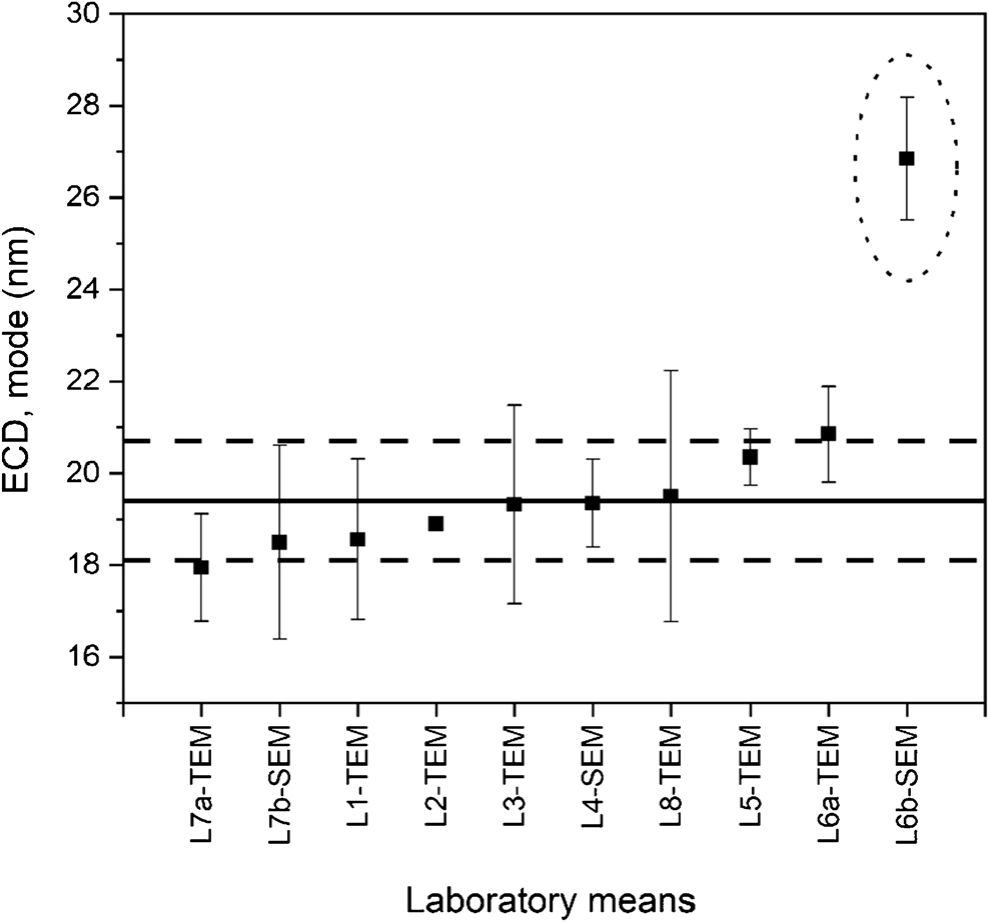
Laboratory mean values of the number-based modal area-equivalent circular diameters (ECD) obtained for QCM (ERM-FD100). The solid line and the set of dashed lines represent the certified value and expanded uncertainty range (*k* = 2). The error bars indicate the expanded (*k* = 2) measurement uncertainties as reported by the laboratories. A significant outlier is surrounded with an ellipse

**Fig. 8:**
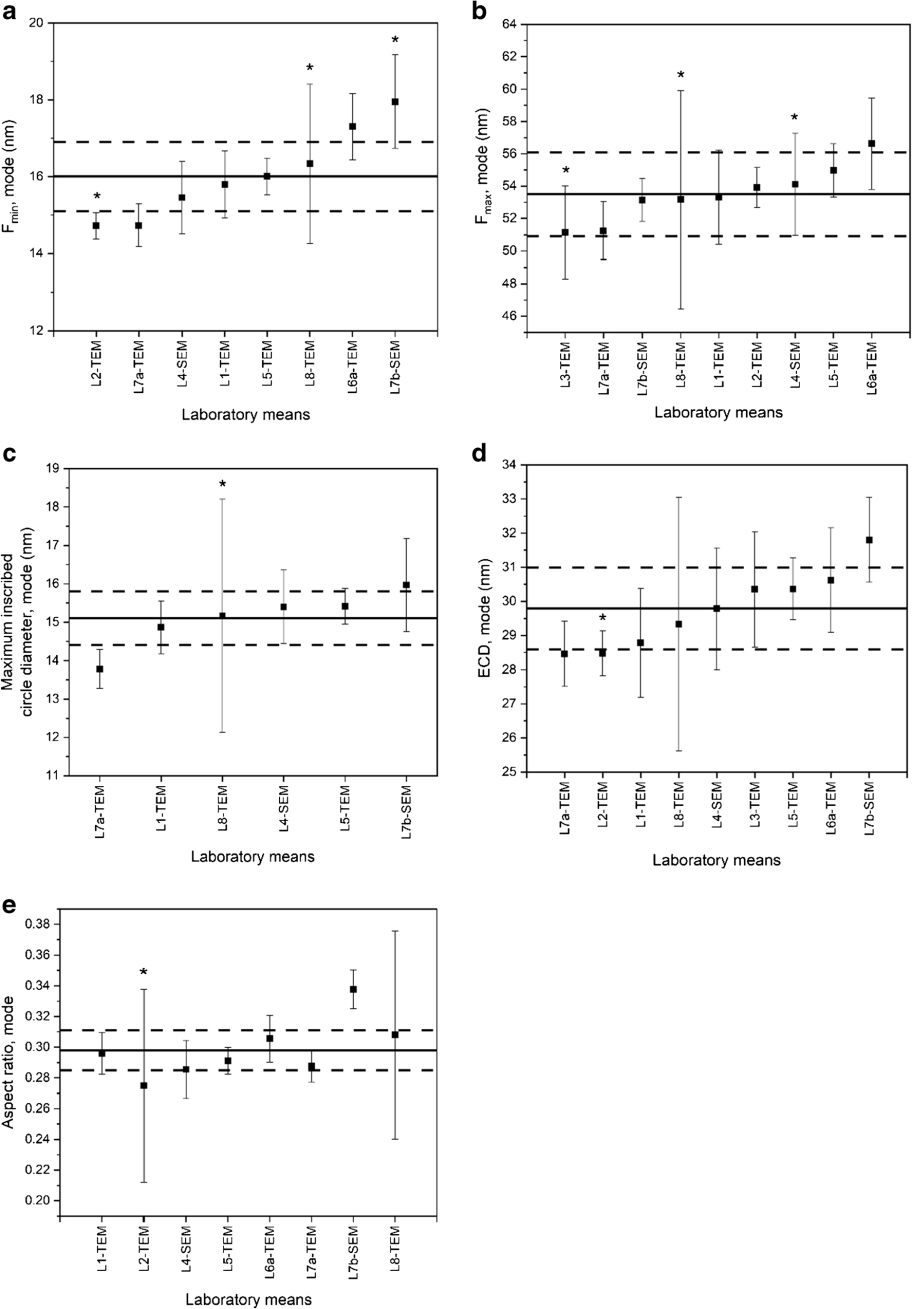
Laboratory mean values of the number-based modal diameters for *F*_min_ (**a**), *F*_max_ (**b**), maximum inscribed circle (**c**) and ECD (**d**) and aspect ratio (**e**), obtained for technically valid datasets of ERM-FD103. The solid lines and the set of dashed lines represent the certified values and expanded uncertainty ranges (*k* = 2). The error bars indicate the expanded (*k* = 2) measurement uncertainties as reported by the laboratories. The asterisk symbols indicate significant variances

The ILC study resulted in ten datasets received from eight different laboratories. Each *laboratory dataset* was composed of measurand-specific sub-datasets listing the six replicate results (three units analysed in duplicate) for a specified measurand, for instance, the modal value of the number-weighed distribution of *F*_min_. The technical evaluation of the datasets comprised of first checking for compliance and adherence to the general ILC protocol (see ESM). It was found that all laboratories followed the ILC protocol, i.e. the specimens were analysed according to the prescribed measurement scheme, the requested target measurands were analysed (Table 1), and reported results were associated with expanded (*k* = 2) measurement uncertainties (error bars in Figs. 7 and 8).

In the next step, the measurement results obtained on the QCM were evaluated statistically using the methodology described in ERM Application Note 1 [37]. As the ILC was used for the characterisation of a *candidate* CRM, the intrinsic reliability of the measurement results obtained on ERM-FD103 could not be easily confirmed, as there was no benchmark to compare with. In that respect, the QCM, which had to be analysed in the same measurement sequence of ERM-FD103, played a key role in validating the ERM-FD103 datasets. The CRM ERM-FD100 was chosen as QCM because the 20 nm nominal diameter of the silica nanoparticles matched the minimum external dimension of the TiO_2_ nanorods. Hence, laboratories that obtained unbiased particle size results for the QCM demonstrated that the applied electron microscopy measurement procedure performed correctly. Based on this evaluation, one laboratory dataset (L6-SEM, Fig. 7) was rejected from the certified value assignment process of ERM-FD103, as the modal particle size result (in terms of ECD) obtained on the QCM was 38% above the QCM’s certified value (solid line in Fig. 7). The other laboratory datasets were all accepted as their modal ECD values agreed statistically with the certified ECD value of the QCM.

Following the rejection of laboratory dataset L6-SEM (Fig. 7), nine laboratory datasets of ERM-FD103 were further evaluated for each measurand of interest (Table 1), except for the maximum inscribed circle diameter for which only seven datasets were received. This was because not all image analysis software programmes used by the participants allowed calculation of the maximum inscribed circle diameter. Also, the results for *F*_min_ and the maximum inscribed circle diameter of one laboratory dataset (L3-TEM, data not shown) were rejected on technical grounds as the laboratory applied an image magnification which was considered inappropriate. At the applied magnification of × 8000, the size of a single data pixel was 3.12 nm. Given the nanorod’s nominal width of 15 nm and the previously mentioned pixel resolution, the particle’s minimum external dimension is represented by about (5.0 ± 0.5) pixels only. Consequently, the minimum external dimension cannot be measured with an uncertainty better than about 10%. As the latter is significantly larger than the relative expanded measurement uncertainty of 5.6% estimated by the laboratory, the reliability of the respective datasets was questioned.

From the laboratory datasets that successfully passed the preliminary quality evaluation, measurand-specific laboratory mean values were calculated from the six replicate results. The laboratory mean values (grouped per measurand, Table 1) were then subjected to further scrutiny using statistical tools such as kurtosis/skewness tests and Grubbs’ and Cochran’s tests, to evaluate the laboratory means for normality and to detect outlying means and variances. All laboratory means followed a univariate normal distribution, as the skewness and kurtosis values, calculated using the dedicated functions in Excel, fell within the range of − 2 and + 2 [40]. The Grubbs’ outlier test did not flag any laboratory mean as statistical outlier on a confidence level of 99%. The retained laboratory mean modal values grouped per measurand are shown graphically in Fig. 8. The error bars correspond to the expanded measurement uncertainties as estimated by the ILC participants. The median values are virtually the same as the modal values and are for that reason omitted in this article. An overview of the median values can be found in the certification report [26].

While no outlying laboratory means were detected, the Cochran’s outlier test did identify outlying variances for a number of laboratory datasets. In Fig. 8, a laboratory mean value accompanied with an asterisk (*) indicates a dataset with a significant variance (*p* < 0.01). It must be noted that the statistical result is independent from the magnitude of the error bars, as the latter represent purely the expanded measurement uncertainties reported by the laboratories. Outlying variances are, however, not a surprise because it is expected that the very different measurement procedures applied by the ILC participants have different intrinsic variabilities. Hence, all retained datasets, including those with outlying variances, were accepted.

For each target measurand (Table 1), certified values were calculated from the retained measurand-specific datasets. Because the laboratories that participated in the ILC study were preliminarily qualified based on demonstrated expertise, and based on the results obtained on the QCM, no weighting of laboratory means was required. Hence, for each measurand, the unweighted mean of laboratory means was assigned as certified value. In Fig. 8, certified values are represented by solid lines. The set of dashed lines represents the certified range (i.e. certified value ± *U*_CRM_). An overview of all individual relative uncertainty contributions, assigned certified values and associated expanded (absolute) uncertainties, *U*_CRM_, is also given in Tables 4 and 5. The relative expanded uncertainties were in the range of 4% (modal ECD) to 6% (modal aspect ratio). These uncertainties are in line with the expanded uncertainties of the certified values for area-equivalent circular diameters of monodisperse colloidal silica [36, 41], indicating that they can be considered realistic and are fit for the intended use of ERM-FD103, which is quality control and length scale calibration of electron microscopes.

As can be seen from the different ILC graphs, the laboratory means of L2-TEM and L7b-TEM (Fig. 8a), L7a-TEM (Fig. 8c) and L7b-SEM (Fig. 8e) hardly overlap with the certified ranges. Their significance at a 95% confidence level was confirmed using the procedure described in ERM Application Note 1 [37]. It must, however, be noted that these significant differences do not necessarily point at a lack of accuracy. The procedure outlined in ERM Application Note 1 is based on comparing the experimental bias (i.e. absolute difference between laboratory mean and the assigned certified value) with the uncertainty of the bias. The latter is estimated by combining the uncertainty of the certified value and the laboratory’s measurement uncertainty. Crucial in this approach is the reliability of the measurement uncertainty estimated by the laboratory. Since CRMs are mostly developed to tackle specific measurement challenges, it is not uncommon that in those situations even expert laboratories sometimes underestimate their measurement uncertainty. As a result, laboratory results that significantly differ from the assigned certified values do not necessarily indicate that the experimental bias is truly significant.

The measurands, or the quantities intended to be measured, of ERM-FD103 are defined by the application of static image analysis as described in ISO 13322-1 [22]. This means that measurement results can only be compared meaningfully if the measurand definition is in full agreement with ISO 13322-1. As all laboratories used SI-traceable calibrants for the calibration of the electron microscopes’ length scales, the certified values assigned to ERM-FD103 are metrologically traceable to the SI unit of length, the metre. Metrological traceability is a property of a measurement result whereby the result can be related to a reference (e.g. an SI unit) through a documented unbroken chain of calibrations [42]. When laboratories use ERM-FD103 for quality control or calibration, then the traceable certified values will provide a solid and direct link between the laboratories’ measurement results and the SI unit of length. Only measurement results that are traceable to the same reference can be compared with each other.

## Conclusions

Over the last decade, JRC has developed and produced a number of CRMs that consist of monomodal and bimodal populations of silica nanoparticles with a near-spherical shape and nominal diameters in the range of 20 to 90 nm. These CRMs have been advantageous for analytical purposes such as validation and quality control of measurement procedures for size analysis of nanoparticles. The effort to produce a CRM of non-spherical nanoparticles, i.e. ERM-FD103, was driven largely by the need to implement recent changes in the EU regulatory framework relative to nanomaterials [5, 6]. The procedures used for the production of ERM-FD103 were consistent with ISO 17034, ISO Guide 35 and ISO/IEC Guide 98-3 [29, 30, 38].

The data presented and discussed in this contribution demonstrate that a TiO_2_ nanorod starting material with tailored properties was successfully transformed into a homogeneous and stable reference material. In addition, the results of an interlaboratory comparison study showed that, for a selection of highly relevant particle size and shape measurands, the use of effectively validated SEM/TEM measurement procedures complemented with a general ILC protocol yields consistent and reproducible results, which served the assignment of a set of 10 certified values. ERM-FD103 has been designed such that it can be integrated into benchmarking and quality control of SEM and TEM measurement procedures with a minimum of effort, i.e. as few as 100 particles need to be analysed. Since the certified values are traceable to the SI unit of length (the metre), and because of the relatively small certified expanded uncertainties, ERM-FD103 can be used also for image and length scale calibration of electron microscopes. Finally, as for any CRM, the certified values provide an effective anchoring point in the traceability network of laboratory results. Only traceable results can be compared worldwide.

## Electronic supplementary material

ESM 1 (PDF 286 kb)
