# Supplementary material for: A new certified reference material for size and shape analysis of nanorods using electron microscopy
Source: Anal Bioanal Chem. 2020 Oct 13;413(1):141–57. doi: 10.1007/s00216-020-02984-z (PMC7801322; doi:10.1007/s00216-020-02984-z)
Supplement: Supplementary file 1 — (PDF 286 kb) [file 216_2020_2984_MOESM1_ESM.pdf]

Analytical and Bioanalytical Chemistry

Electronic Supplementary Material

A new certified reference material for size and shape analysis of nanorods using electron microscopy

Vikram Kestens, Tsvetelina Gerganova, Gert Roebben, Andrea Held

1) Interlaboratory comparison measurement protocol

Aim and scheme of the ILC study

The aim of this interlaboratory comparison (ILC) study is the characterisation and certification of a titanium dioxide (TiO_2) nanorod candidate certified reference material (CRM; labelled as ERM-FD103) by SEM and TEM. The basis of this certification approach is the randomisation of the (unknown) laboratory biases. This randomisation is only successful if the measurements within a laboratory are performed under intermediate precision conditions and if the collected results are independent. If one laboratory applies several methods then, these analyses can be treated as if they came from independent laboratories, provided instruments are calibrated differently, and test specimens are prepared separately.

Each contractor will receive three ampoules of the candidate CRM (ERM-FD103), each containing a nominal mass fraction of 1 g/kg TiO_2 nanorods dispersed in about 2 mL of *n*-butanol.

In addition to the candidate CRM, each contractor will receive one ampoule of a quality control material (QCM) that consists of about 9 mL of a suspension of near-spherical silica (SiO_2) nanoparticles with an average particle size in the range of 10 nm to 30 nm and a nominal mass fraction of 10 g/kg.

In total, a minimum of eight independent specimens shall be prepared and analysed:

QCM (1 ampoule x 2 replicates) for the following particle **size** measurand:<br><br>- **Area-equivalent diameter (modal value)**	2 specimens
Candidate CRM (3 ampoules x 2 replicates) for the following particle **size** and **shape** measurands:<br><br>- **Area-equivalent diameter (mode, median)**<br>- **Minimum Feret diameter (F_{min})**<br>- **Maximum Feret diameter (F_{max})**<br>- **Aspect ratio (as reported by software) and calculated as F_{min}/F_{max}**<br>- **Maximum inscribed circle**	6 specimens
**Total**	**8 specimens**

The term 'replicate' shall be interpreted as an SEM or TEM specimen. The latter shall be prepared by transferring a representative portion of the TiO_2 nanorods from the ampoule to an appropriate flat SEM or TEM substrate.

The ILC study is organised as follows:

- for each awarded contract, JRC dispatches three ampoules of the candidate CRM and one ampoule of the QCM to the contractor;
- the contractor shall analyse the candidate CRM and QCM according to the instructions provided and report within **8 weeks** after the date of receipt of samples (based on DHL tracking information);
- JRC evaluates and analyses the received datasets using appropriate statistical techniques;
- financial settlement of the order(s) by JRC;
- results of the ILC study are used in the certification report where they will be presented in an anonymous manner.

Specimen preparation

1) Before opening an ampoule, the ampoule must be gently inverted several times to ensure the homogeneity of the suspension and to re-suspend possibly settled particles. If after homogenisation some of the suspension is still present in the upper part (bulb-like head) of the ampoule, it can be removed by gently flicking the bulb-like head with the forefinger while tilting the ampoule. The ampoule is pre-scored and can be opened by applying moderate pressure with one's thumb to snap off the ampoule's head. Contents of an ampoule must be used the same day as opened. After opening of the ampoule, the ampoule should be either closed with paraffin film or the content should be transferred to a clean glass vial that can be closed with an appropriate cap.

2) The TiO_2 particles, which have external dimensions in the range of 1 nm to 100 nm, should be analysed as-received (i.e. without filtration, centrifugation or sonication prior to analysis). In case dilution is required, ***n*-butanol anhydrous, purity ≥ 99.8 %**, must be used.

3) Aliquots shall be taken from the ampoule using clean pipette tips and avoiding to touch the edges of the ampoule. A new (unused) pipette tip shall be used for each aliquot.

4) In avoiding contamination of the test specimens, it is highly recommended to prepare the SEM or TEM specimens in a low contamination environment (i.e. a clean room or a laminar flow bench).

5) The deposition of TiO_2 nanorod particles onto a suitable SEM or TEM substrate shall be based on the **contractor's own established specimen preparation procedure**. The applied procedure should ideally generate a uniform distribution of particles across the entire substrate without excessive amounts of agglomerates and touching particles being formed. In particular for particle shape measurements, the used substrate shall be **flat** over the selected field of view, uniform, and provide good contrast between particles and background.

6) If the minimum required number of particles cannot be observed and measured using one SEM or TEM specimen, then the contractor shall prepare additional specimens from the same ampoule until the minimum required number of particles can be measured.

7) Opening and preparation of test specimens shall be performed according to the measurement scheme as shown below.

**Day 1**	**Day 2**	**Day 3**
QCM colloidal silica Specimen 1	Candidate CRM Ampoule ID … Specimen 1	Candidate CRM Ampoule ID … Specimen 1
Candidate CRM Ampoule ID … Specimen 1	Candidate CRM Ampoule ID … Specimen 2	Candidate CRM Ampoule ID … Specimen 2
Candidate CRM Ampoule ID … Specimen 2		
QCM colloidal silica Specimen 2	-	

Calibration

1) The contractor shall ensure that the performance of the electron microscope is regularly verified and that it is calibrated to the SI unit of length (metre) at operating conditions similar to those to be used for the analysis of the QCM and the candidate CRM.

2) Calibration materials used must fulfil all requirements of a certified reference material, i.e. they must be homogeneous, stable, and come with a certified value with associated uncertainty and metrological traceability statement.

3) A combined relative standard uncertainty (at a confidence level of 68 %) that is associated to the calibration coefficient of the instrument's length scale shall be estimated and provided together with a detailed description of the applied calibration procedure, in the analysis report.

Image acquisition

1) Images shall be captured using an electron microscope combined with a suitable detector, resulting in the generation of count- or number-weighted particle size/shape distributions.

2) The magnification used for the QCM and candidate CRM shall be large enough to measure the largest external dimension of the nanorods while having sufficient resolution to also accurately measure the rod's smallest external dimension.

3) For particle shape measurements, a higher pixel resolution might be necessary than for particle size measurements. It is, however, up to the user to set the optimal acquisition parameters and pixel resolution and to prove that they were chosen properly.

4) For each specimen prepared from the QCM and the candidate CRM, at least **250 individual** (non-touching and not agglomerated) particles shall be imaged and analysed (see Section 5.1) and analysed. If the measurement protocol does not generate sufficient number of individual particles, then the contractor shall measure at least **500 touching** (**but not overlapping!**) particles. The measured particles shall originate from at least **five** different fields of view (i.e. images). If the required number of particles cannot be reached using five images, then additional images shall be captured until the minimum number of particles is reached. For a given specimen, the different images shall be captured randomly, but widely separated from each other, across the entire surface of the specimen surface.

5) The specimen surface at the field of view must be **flat**. This is particularly important for particle shape measurements, i.e. where a tilt angle can significantly affect the accuracy of the measurement result obtained on the longest external particle dimension.

Image analysis (according to ISO 13322-1:2014) and statistics

1) For each QCM specimen, at least **250 individual and non-touching** particles (or **500 touching** but not overlapping particles – see Section 4.4) from at least five different images shall be analysed with respect to the **area-equivalent circular diameter**. Foreign artefacts (e.g. contamination and dust particles, residues from drying, etc.) as well as particles cut by the measurement frame must be excluded from the data analysis process. In case the contractor decides to analyse touching particles rather than individual non-touching, particles, an appropriate particle separation protocol must be applied and all images showing the detection and separation of particles shall be provided.

2) For each QCM specimen, the obtained raw results must be graphically represented in a histogram (density distribution q_0 according to ISO 9276-1:1998) having a linear abscissa (x-axis). The modal result of this number-weighted particle size distribution shall be reported along with the associated relative (%) expanded measurement uncertainty (at a confidence level of 95 %). The optimal number of size bins for the density distribution shall be defined by the contractor.

3) For each candidate CRM specimen, at least **250 individual** (non-touching and not agglomerated) particles, or **500 touching** but not overlapping particles, from at least five different images shall be analysed with respect to the following measurands:

- Minimum Feret (F_{min}) diameter according to ISO 9276-6:2008;
- Maximum Feret (F_{max}) diameter according to ISO 9276-6:2008;
- Aspect ratio as calculated by the user's software, including an unambiguous description of the parameters used (and/or equation) **and** as the ratio of F_{min}/F_{max};
- Area-equivalent diameter according to ISO 9276-6:2008;
- Maximum inscribed circle.

Foreign artefacts (e.g. contamination and dust particles, residues from drying, etc.) as well as particles cut by the measurement frame must be excluded from the data analysis process.

4) For **each measurand** and for each specimen prepared from the candidate CRM, raw measurement results must be graphically represented in a **histogram** (i.e. as a density distribution q_0 having a linear abscissa) and as a **cumulative distribution** (Q_0). The binarisation process shall be optimised and defined by the contractor. The contractor shall report the modal value of the density distributions q_0 and the median value from the cumulative distributions Q_0. The median shall be calculated across the following particle size ranges:

- Area-equivalent circular diameter: 5 nm to 80 nm

- F_{min}: 5 nm to 35 nm

- F_{max}: 20 nm to 80 nm

- F_{min}/F_{max}: 0.1 tot 0.5

All results shall be reported along with the associated relative (%) expanded measurement uncertainties (at a confidence level of 95 %).

Reporting

1) A detailed and signed analysis report shall be sent by post mail or courier service to the study coordinator within **8 weeks** after the date of receipt of samples. The analysis report shall contain as a minimum the information listed in the below mentioned table.

**Generic information** *(according to ISO/IEC 17025)*
Name and address of the laboratory, and location where tests were carried out, if different from the address of the laboratory
Name and address of the client
Unique identification of the analysis report, repeated on each page of the report
Page numbering indicated as e.g., “Page 1 of 15”
Operator’s name
Name, function and signature of persons authorising the analysis report
**Sample information** *(according to ISO 13322-1)*
Date of receipt of the candidate CRM and QCM samples
Identification of the laboratory samples and, if relevant, identification assigned to the ampoules by the contractor
Date when the ampoules were opened and specimens were prepared
Identification of the specimens (e.g., ampoule#_replicate#)
Complete description of the method used for sub-sampling, if required, and SEM/TEM specimen preparation, with full quantitative details of the nominal mass, volumes and compositions of

products, in case dilution was applied
Type of the used sample holder/substrate
**Sample preparation**
Dilution and dilution medium
Sample volume intake
Sample preparation/drying
Sample grid/stub/sample holder (mesh size, coating, copper/gold/mica)
**Method and instrument information**
Make and type of the electron micoscope
Date of the last instrument performance check/maintenance
Description of the image magnification calibration procedure, including description of the used calibrant(s)
Description of the method used (magnifications, CCD camera, nominal camera length, acceleration voltage, tilt angle, spot size, aperture, etc.)
**Image analysis and results**
Date of performance of the tests
For non-touching particles: at least one representative raw micrograph and one annotated micrograph used for particle identification and analysis per specimen<br>For touching particles: all raw micrographs and annotated micrographs
For each specimen, and for each measurand (F_{min}, F_{max}, aspect ratio, F_{min}/F_{max}, area-equivalent diameter and maximum inscribed circle), one density distribution and one cumulative distribution based on at least 250 individual particles or 500 touching particles.<br>All particle dimension measurands shall be reported in nanometre (nm).
Characteristic values from number-weighted distributions to be reported:<br>Density distribution (linear abscissa): modal value and arithmetic mean<br>Cumulative distribution: median, 25 % and 75 % percentiles
Pixel size/resolution (nm)
Micrograph size (μm)
Total area imaged per sample (μm^2)
Counting and analysis procedure and number of counted/analysed particles
Binning procedure
Description of the estimation of the mode (normal/Gaussian fitting, highest bin, etc.)
Estimation of the measurement uncertainty associated to the number-based modal and median particle size and aspect ratio values (see Section 6.2)
Description of the image analysis software package used
Description regarding adjustment of contrast, brightness, greyscale threshold, etc.
Description of the aspect ratio parameter reported by the applied image analysis software
Description regarding the usage of image filters (smoothing, NxN, mean, median)
Description regarding the image analysis protocol dealing with the separation and identification of touching particles (manually/automatically discard all touching particles, manual or automatic particle separation filters, morphology threshold based separation, etc.)

2) The contractor shall provide an estimation of a realistic relative (%, nm/nm) expanded measurement uncertainty (confidence level of about 95 %) associated to a single measurement result (i.e. the average of the results obtained on ≥ 250 (or ≥ 500) particles per specimen and for a given measurand), and details on how this uncertainty was derived.

Ideally, the relative expanded measurement uncertainty, U, should be estimated from method validation data. If such data is available, individual relative standard (confidence level of 68 %) uncertainties estimated for method repeatability (u_r), day-to-day variation or intermediate precision (u_{ip}), instrument calibration (u_{cal}) and method

trueness (u_t) can be combined in order to arrive at a combined standard measurement uncertainty, u_{meas} (see equation below). For a confidence level of 95 %, u_{meas} is multiplied with a coverage factor (k = 2) resulting into a relative expanded measurement uncertainty, U.

$$u_{meas} = \sqrt{u_r^2 + u_{ip}^2 + u_{cal}^2 + u_t^2}$$

$$U = u_{meas} \times k$$

In case method validation data is not available, u_{meas} and U, may be estimated following alternative approaches, such as:

- in-house quality control charts
- results obtained during previous ILC studies
- expert judgement

2) Measurement procedures used during the ILC study

Table S1 Overview of SEM and TEM measurement procedures used by the ILC participants (from: Gerganova T, Roebben G, Kestens V. The certification of size and shape parameters of titanium dioxide nanorods in 1-butanol solution: ERM-FD103. Certification report EUR 29781. Luxembourg: Publications Office of the European Union; 2019)

Laboratory Code	Specimen preparation	Instrument	Calibration	Image acquisition	Image analysis and evaluation
L1-TEM	The as-received material was 5x diluted in 1-butanol. 15 μL of the diluted suspension was brought onto a pioloform® carbon-coated 400 mesh Cu grid. The grid was left in contact with the suspension for 10 min while being covered with an empty petri dish to limit evaporation of 1-butanol. After the incubation period, the grid was blotted dry to remove excess sample and left to air-dry at room temperature.	FEI Tecnai Spirit	Optical diffraction cross-grating with 2160 lines/mm and 463 mm line spacing (Agar Scientific)	The TEM instrument was operated at 120 kV and at a spot size of 3. Micrographs were captured using a bottom-mount 4k x 4k Eagle CCD-camera and at a 30,000x magnification.<br>Pixel size = 0.37 nm	AnalySIS Solution of iTEM (Olympus Soft Imaging Solutions GmbH)<br>Automatic contrast/brightness correction, manual selection of threshold for particle detection based on mass-thickness contrast, 10x10 filter for reducing background noise, particle detection filters 0-40 nm (min diameter), 10-50 (ECD), 0-0.8 (sphericity), 0.6-1 (convexity) and 0-0.8 (shape factor).<br>Number-based PSDs were iteratively fitted with a Gaussian function.
L2-TEM	For each CRM unit, replicates 1 were prepared by bringing a 10 μL drop of the undiluted as-received material onto a carbon-coated 200 mesh Cu grid. Replicates 2 were dipped into the undiluted suspension. Grids were allowed to in an ISO class 5 clean bench.	Philips CM120	MAG*I*CAL® (Electron Microscopy Sciences), traceable to SI Unit of length, metre, through interplanar lattice spacing of a silicon crystal.	The TEM instrument was operated at 100 kV, at a spot size of 1 and a filament emission of 2. Micrographs were captured using an Optronics 2048 x 2048 pixel CCD camera.<br>Pixel size = 0.42 nm	ImageJ (National Institute of Health)<br>Contrast/brightness correction, no image filters were utilised, non-touching and touching particles were manually sized using the “Polygon” selection tool.<br>The modal values correspond to the highest bins in the number-weighted PSDs.

Laboratory Code	Specimen preparation	Instrument	Calibration	Image acquisition	Image analysis and evaluation
L3-TEM	Aliquots of about 200 μL were taken from the undiluted material and transferred onto carbon vaporised 200 mesh Cu grids. Immediately after deposition, the bottom of the grids was carefully wiped on a filter to remove supernatant fluid by capillary attraction. No particle coating was applied.	Hitachi H-7500	MAG*I*CAL® serial no. 1310 (Norrox Scientific Ltd. Ontario), traceable to SI Unit of length, metre, through the calibrated spacing of a group of five lines (max. distance of 108.5 nm)	The TEM instrument was operated at 100 kV and at a spot size of 5 (4 μm). Micrographs were captured using a Tengra (EMSIS) 2304 x 2304 pixel CCD camera and at 8,000x to 10,000x magnifications. Pixel size = 3.12 nm	AnalySIS Solution of iTEM (Olympus Soft Imaging Solutions GmbH) Adjustment of grey-scale threshold, no image filters were utilised, separation and identification of particles was accomplished manually with a hand drawn one pixel line. Particles cut by the image edge and overlapping particles were manually deleted.
L4-SEM	Samples were diluted into 10x *n*-butanol. 5 μL aliquots of the dilute suspension were drop-cast on single-crystal silicon chips and were let to dry. Specimens were imaged as deposited; a conductive coating was not required.	FEI Helios Dual-Beam	NanoLattice™ standard with nominal 100 nm pitch (VLSI Standards, Inc.). Pitch size standard calibrated on metrological AFM, pitch value of 99.98 nm ± 1.5 nm (k = 2)	The SEM instrument was operated at 15 keV and at a beam current of 43 pA. Micrographs were captured at a magnification of 300,000x. Pixel size = 0.6 nm	ImageJ version 1.51 (National Institute of Health) Contrast and brightness were set so that the histogram covered 80 % of the grey levels without over or under saturation. Particle boundaries were selected manually. Touching but not overlapping particles were manually selected for image analysis. Number-weighted histograms of F_{max} and ECD were fitted with Weibull distributions, histograms of F_{min} and aspect ratio were fitted with Gaussian functions.

Laboratory Code	Specimen preparation	Instrument	Calibration	Image acquisition	Image analysis and evaluation
L5-TEM	5 µL aliquots of the undiluted as-received material were brought onto carbon-coated 200 mesh Cu grids.	Philips CM200	Optical diffraction cross-grating (No. 673) with 2000 lines/mm and 500 nm line spacing (Ted Pella, Inc.)	The TEM instrument was operated at 80 kV and at a spot size of 1. Micrographs were captured using an EMSIS Megaview GR CCD camera and at 150,000x magnification.<br>Pixel size = 0.37 nm	Image PRO Plus 7.0 (Media Cybernetics)<br>Contrast and brightness were manually set, no image filters were utilised. For each specimen, 250 non-touching particles were measured manually using the "Polygon" selection tool.
L6a-TEM	100 µL of the undiluted material was taken and diluted 10x into *1*-butanol. 5 µL aliquots of the diluted sample were brought onto Formvar® carbon-coated 300 mesh Cu/Rh grids. The specimens were dried under an IR lamp for 15 min and then kept in a closed petri dish prior to analysis.	FEI Tecnai G2	Optical diffraction cross-grating (09202-AB) with 2160 lines/mm and 463 mm line spacing (SPI Supplies)	The TEM instrument was operated at 200 kV and at a spot size of 3. Micrographs were captured using WA-Veleta camera and at 390,000x magnification.<br>Pixel size = 0.14 nm	Aphelion Dev (ADCIS)<br>Automatic adjustment of grey-scale threshold, select dark objects, split touching objects, fill holes, remove object touching the frame, Gaussian filter of 7 pixels applied.<br>Modal values of histograms were determined from Gaussian fits.
L6b-SEM	100 µL of the undiluted material was taken and diluted 20x into *1*-butanol. The diluted samples were treated with an Heidolph ultrasonic head at 100 % for 2 min. Samples were maintained at low temperature by immerging sample vials into an ice/water bath. 5 µL aliquots of the diluted material were brought onto Formvar® carbon-coated 300 mesh Cu/Rh grids. The specimens were dried under an IR lamp for 15 min and then kept in a closed petri dish prior to analysis.	JEOL 7500F	Optical diffraction cross-grating (09202-AB) with 2160 lines/mm and 463 mm line spacing (SPI Supplies)	The SEM instrument was operated at 15 kV and at a beam current of 20 µA. Micrographs were captured at a magnification of 200,000x.<br>Pixel size = 0.47 nm	Aphelion Dev (ADCIS)<br>Automatic adjustment of grey-scale threshold, select bright objects, split touching objects, fill holes, remove object touching the frame, Gaussian filter of 5 pixels applied.<br>Modal values of histograms were determined from Gaussian fits.
L7a-TEM	About 800 µL of the undiluted as-received material was transferred to	JEOL JEM-2100F	MAG*I*CAL® serial no. 1426, traceable to SI	The TEM instrument was operated at 200 kV and at a	Image PRO Plus 7.0 (Media Cybernetics)

Laboratory Code	Specimen preparation	Instrument	Calibration	Image acquisition	Image analysis and evaluation
	an Eppendorf. A plasma-treated Formvar® carbon-coated 200 mesh Cu grid was then submerged in the undiluted suspension for several hours. The specimen was air-dried in a contamination-free environment.		Unit of length, metre, through interplanar lattice spacing of a silicon crystal.	spot size of 1. Micrographs were captured at 40,000x magnification.<br>Pixel size = 0.41 nm	Automatic adjustment of grey-scale threshold, a 3x3 median image filter was utilised. The built-in Watershed Split and Auto Split functions were used to separate and identify touching particles. Manual separation of touching but not overlapping particles was applied for those particles which could not be separated automatically.<br>Histograms were evaluated using MATLAB R2017b
L7b-SEM	About 800 μL of the undiluted as-received material was transferred to an Eppendorf. A plasma-treated Formvar® carbon-coated 200 mesh Cu grid was then submerged in the undiluted suspension for several hours. The specimen was air-dried in a contamination-free environment.	JEOL JSM-6500F	Standard for SEM calibration, serial no. S170A (Agar Scientific), certified value 461.2 nm ± 1.2 nm (k = 2)	The SEM instrument was operated at 15 kV and at a spot size of 6. Micrographs were captured at a magnification of 120,000x.<br>Pixel size = 0.78 nm	Image PRO Plus 7.0 (Media Cybernetics)<br>Automatic adjustment of grey-scale threshold, a 3x3 median image filter was utilised. The built-in Watershed Split and Auto Split functions were used to separate and identify touching particles. Manual separation of touching but not overlapping particles was applied for those particles which could not be separated automatically.<br>Histograms were evaluated using MATLAB R2017b
L8-TEM	Samples were diluted into 200x *1*-butanol. 6 μL aliquots of the diluted material were drop-cast onto a Lacey carbon-coated 400 mesh Cu grids. The specimens were dried in a cytotoxic cabinet for > 6 hr.	JEOL 2100	Gold nanoparticle reference material NIST 8013 (NIST)	The TEM instrument was operated at 200 kV and at a spot size of 1. Micrographs were captured using a Gatan Ultrascan 1000 CCD camera at 56,000x magnification.<br>Pixel size = 0.26 nm	ImageJ version 1.50f (National Institute of Health)<br>Semi-automated particle detection, manual setting of contrast, brightness and grey-scale thresholding applied 7 x 7 median image filter was used. Individual particle outlines were measured manually. All

Laboratory Code	Specimen preparation	Instrument	Calibration	Image acquisition	Image analysis and evaluation
					particles cut by, or touching, the measurement frame were excluded.<br>The modal values correspond to the highest bins in the number-weighted PSDs.
